# Spica cast as an alternative to general anesthesia for lower limb MRI in young children

**DOI:** 10.1007/s10195-013-0251-1

**Published:** 2013-06-25

**Authors:** P.-Y. Rabattu, A. Courvoisier, E. Bourgeois, A. Eid, C. Durand, J. Griffet

**Affiliations:** 1 Department of Paediatric Orthopedic Surgery, Hopital Couple Enfants, Joseph Fourier University, BP 217, 38043 Grenoble Cedex 9, France; 2 Department of Paediatric Radiology, Hopital Couple Enfants, Joseph Fourier University, Grenoble, France

**Keywords:** Spica cast, MRI, Lower limb, Children

## Abstract

**Background:**

The conventional approach for MRI procedures in very young children is to use general anesthesia which comes with inherent risks. Non-pharmacological strategies to reduce anxiety in children have also been described, but they all require patient cooperation. The purpose of the study was to evaluate the ability to complete diagnosis using temporary spica cast immobilization (TSCI) in children less than 3 years old undergoing MRI procedures for lower limb disorders.

**Materials and methods:**

A retrospective review identified 14 children under 3 years old that had required an MRI for a lower limb disorder, using TSCI. The MRI procedure was performed for evaluation of hip dysplasia, bone infections, limping, evaluation of soft tissue tumor and femoral head osteonecrosis. A spica cast was fitted by the pediatric orthopedic team. The MRI procedure was subsequently performed.

**Results:**

Diagnosis was achieved in all cases. The radiologist identified movement artifacts (14 %) that did not impair the image quality enough to prevent interpretation.

**Conclusion:**

TSCI is a safe, effective and costless procedure avoiding general anesthesia for young patients under 3 years old who require MRI for pelvis or lower limb disorders.

**Level of evidence:**

IV.

## Introduction

MRI has become the imaging reference for lower limb bone and cartilage disorders [1–3]. Applying MRI procedures to small children is hampered by the combination of patient noncooperation, rapid or irregular respiratory rate, and small-scale anatomy, meaning that very small movements can create motion artifacts that render images non-diagnostic. The conventional approach in very young children is to use general anaesthesia (GA). As with all GAs, this comes with inherent risks [4]. Whilst serious adverse effects of GA are rare, less serious adverse effects occur in approximately 0.4–1.5 % of cases [4]. Non-pharmacological strategies to reduce anxiety in children in preparation for MRI have also been described. These can broadly be categorized as play-based therapy [5], desensitization and cognitive behavior therapy [6–8]. The use of a mock MRI service is also effective in reducing GA rates [9]. But most of these procedures have been described in children older than 3 years as they require patient cooperation.

A procedure of temporary spica cast immobilization (TSCI) was developed to reduce the rate of GA in children less than 3 years old undergoing MRI procedures for lower limb disorders. The purpose of the study was to evaluate the ability to complete diagnosis using the procedure of temporary spica cast immobilization in children less than 3 years old undergoing MRI procedures for lower limb disorders.

## Materials and methods

A retrospective review identified 14 children under 3 years old that had required an MRI for a lower limb disorder, using the TSCI procedure. The median age was 16 months [range: 6–36 months]. The indications for the MRI procedure are reported in Table 1.

**Table 1:** Clinical cases and MR findings

Case	Age (months)	Side	MRI indication	MRI findings	Artifact
1	13	Left	Inguinal cellulite	Inguinal cellulite without underlying bone infection	No
2	30	Right	Ankle pain, fever, normal US	Tibial osteomyelitis	No
3	34	Right	Suspected femoral head osteonecrosis	Femoral head osteonecrosis	No
4	17	Left	Knee pain, fever	Knee arthritis without bone involvement	No
5	15	Right	Suspected popliteal cyst	Popliteal cyst	No
6	36	Right	Knee pain, fever	Femoral osteomyelitis	No
7	18	Left	Suspected osteoarthritis of the hip	Femoral head epiphysis infection	Yes
8	6	Left	Hemi-hypertrophy, foot edema	Benign vascular tumour of the foot	Yes
9	23	Right	Foot cellulite and positive scintigraphy	Normal	No
10	32	Left	Foot cellulite and positive scintigraphy	First metatarsal osteomyelitis	No
11	22	Left	Chronic hip pain, no fever	Greater trochanter osteomyelitis	No
12	18	Right	Chronic limping, no fever, normal X-rays	Knee arthritis without bone involvement	No
13	24	Right	Knee pain, no trauma, no fever	Normal	No
14	30	Right	Hip pain, no fever, US: hip effusion	Hip arthritis without bone involvement	No

*US* ultra sound

### Temporary spica cast immobilization procedure (TSCI)

**Fig. 1:**
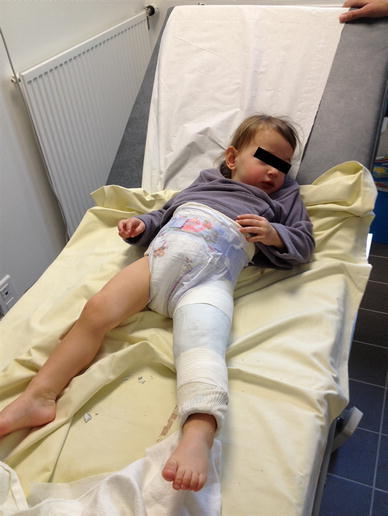
Photograph of a 2-year-old girl after temporary spica cast immobilization before MRI for great trochanter chronic pain

The day of the MRI procedure, each child had an appointment in the pediatric orthopedic department. A spica cast was fitted by the pediatric orthopedic team used to the technique of spica cast immobilization (Fig. 1). All casts were standard plaster casts. The cast was either fitted in a standing position or on a spica cast table, depending on the pain. Fitting the cast in a standing position is usually easier and quicker. Only the affected limb was fitted with a cast. The foot was only included in the cast when the location of the disorder was distal to the knee. The mean time which elapsed from cast application to the MRI procedure was 75 min [range 60–130]. The MRI procedure was subsequently performed (Philips-Achieva 3.0T X-series MRI) according to the conventional sequences and radiofrequency coils used for bone and joint disorders. The following set of sequences was performed depending on the location: T1-weighted and T2-weighted with/without fast-spin echo and/or short tau inversion recovery. Intravenous contrast material was used in cases where bone viability needed to be assessed, in cases of suspected infection or in bone and soft-tissue tumors. Parents stood close to their child during the whole examination. The spica cast was removed immediately after the MRI procedure if necessary. All MRI were examined by a consultant radiologist to determine whether the diagnosis could be reached or if the rate of movement artifacts were too numerous, hampering clinical scan interpretation.

## Results

**Fig. 2:**
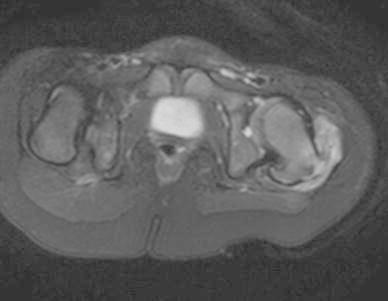
Sample good quality MRI image (T1 gadolinium). A chronic osteomyelitis of the left great trochanter was diagnosed

Diagnosis was achieved in all cases. MRI findings are reported in Table 1. An example of an MRI image of a left great trochanter osteomyelitis is illustrated in Fig. 2. In two cases (cases 7 and 8), the radiologist identified movement artifacts (14 %) that did not impair the image quality enough to prevent interpretation. Afterward, we realized that the cast, in these two cases, was not stiff enough, particularly at the hip junction. No complication was encountered.

## Discussion

The purpose of the study was to evaluate the ability to reach diagnosis with MRI for lower limb affections using the procedure of TSCI in children younger than 3 years old. The results show that diagnosis was achieved in all cases despite movement artifacts in two cases. GA was avoided in all cases.

TSCI has become a standard procedure in our institution. Historically, the first MRI procedures under spica cast immobilizations were performed during the follow-up of babies treated for congenital dislocation of the hip. In those cases, the plaster cast was usually fitted under GA in the operating room. The MRI procedure was subsequently performed to analyze the position of the femoral head and the acetabulum morphology. This procedure has been recently described without GA [10]. We extended the procedure to infections, tumors and other lower limb disorders, but with temporary cast immobilization. For children that could stand painlessly on their legs, the cast was done in a standing position, which was quicker and more tolerable. When the standing position was not possible, the spica cast was fitted using the usual spica table. In each case, only the lower limb affected was put into a cast. Ideally, the patient should be managed on an outpatient basis, and many patients requiring TSCI were managed without any hospital stay. GA is now avoided for most lower limb disorders in small children in our institution. Thus, the main limitation of this study is its retrospective design with no control group using GA.

There is practically no limitation of the TSCI. The younger the child, the more efficient plaster cast immobilization is. Older patients may cry or move the trunk, but most children sleep during the MRI procedure. In this series, only the affected limb was put in a cast, but it is still possible, although time consuming, to cast both sides if the child is moving too much. In two cases, where the cast was too soft, artifact movements were observed but did not impair the MRI procedure. Therefore, the cast should be fitted with a team used to the technique of spica cast immobilization. The only limitation is the topography of the disorder. The whole lower limb and the pelvis may be explored with this procedure but above the sacroiliac joints the risk of movement artifacts is too great.

In this work, the delay between cast application and MRI was at least 60 min. This time may be not sufficient to allow the plaster cast to dry completely, but the water remaining in the plaster cast did not alter the quality of the MRI images. TSCI is a safe, effective and costless procedure avoiding GA. In our institution, TSCI has become the gold standard for young patients under 3 years old who require MRI for pelvis or lower limb disorders.
